# Bridging the intention-behavior gap in physical activity among pregnant women with gestational diabetes mellitus: a qualitative study of self-control strategy use and dynamics

**DOI:** 10.1186/s12966-026-01923-y

**Published:** 2026-04-20

**Authors:** Qianru Liu, Fang Xie, Zheshuai Yang, Pingping Guo, Rujia Zhao, Ying Jin, Xiaojuan Wang, Wei Zhang, Shu Li, Hengchang Liu, Suwen Feng

**Affiliations:** 1 Women’s Hospital, School of Medicine, Zhejiang University, No.1 Xue Shi Road, Hangzhou, Zhejiang Province 310006 China; 2 Faculty of Nursing, School of Medicine, Zhejiang University, Hangzhou City, Zhejiang Province China; 3 School of Management, Zhejiang University, Hangzhou City, Zhejiang Province China; 4 Department of Obstetrics and Gynecology, Chinese People’s Liberation Army General Hospital, Beijing City, China

**Keywords:** Self-control, Physical activity, Gestational diabetes mellitus, Qualitative study

## Abstract

**Background:**

Although pregnant women with gestational diabetes mellitus (GDM) often intend to engage in physical activity (PA), they may struggle to translate these intentions into action. Self-control could support PA goal pursuit among this population, as it enables individuals to prioritize health goals over immediate rewarding temptations, such as the urge to rest or sedentary leisure. However, the role of self-control has received limited attention in the existing studies. Therefore, this study aimed to explore how pregnant women with GDM use self-control to bridge the gap between PA intention and behavior in daily life.

**Methods:**

A descriptive qualitative study was conducted. 24 pregnant women with GDM were recruited using purposive sampling at a tertiary hospital in Hangzhou City, Zhejiang Province, China, from April to May 2025. Data were collected through semi-structured interviews and analyzed using qualitative content analysis.

**Results:**

Three themes were identified: conflict between the pursuit of PA goals and temptations, context-adaptive self-control strategies for PA, and dynamics of self-control for PA during pregnancy. Despite strong PA intentions driven by concerns for maternal and fetal health, pregnant women with GDM reported conflicts between their goals and temptations, with rationalization of temptations emerging as a key psychological process linked to reduced PA engagement. To manage this conflict, pregnant women with GDM employed four types of self-control strategies: internal drive strategies based on health cognition and self-motivation, autonomous construction of external supervision strategies, technology-enabled strategies, and behavioral automation strategies. Self-control for PA was found to be dynamic in response to metabolic, physical, and psychosocial contexts, characterized by feedback-driven goal adjustment, strategies switching, self-control failure coping, and self-control motivation fluctuation throughout pregnancy.

**Conclusions:**

This study provides a self-control perspective to better understand and promote PA engagement in pregnant women with GDM. Healthcare providers may consider supporting these women to identify and reappraise rationalizations of temptations, develop a personalized repertoire of self-control strategies with technological support, and foster self-control motivation for PA habit formation.

**Supplementary Information:**

The online version contains supplementary material available at 10.1186/s12966-026-01923-y.

## Background

Gestational diabetes mellitus (GDM) is one of the most common metabolic complications during pregnancy, affecting approximately 14% of pregnant women worldwide [1]. It not only increases the risks for cesarean delivery, preterm birth, and infant complications such as macrosomia, neonatal respiratory distress syndrome, and neonatal jaundice [2], but also imposes a substantial public health burden, with the annual diagnosis and treatment costs reaching USD 5.5 billion in China and USD 1.6 billion in the USA [3]. Strengthening GDM management has therefore become a global public health priority to safeguard maternal and neonatal health and reduce socioeconomic costs.

Physical activity (PA), defined by the World Health Organization (WHO) as “any bodily movement produced by skeletal muscles that results in energy expenditure” [4], is a core nonpharmacological treatment for GDM. Regular PA can improve glycemic control, reduce the need for insulin therapy, alleviate negative emotional states, and decrease the risk of pregnancy-related complications [5]. Despite international guidelines recommending at least 150 min of moderate-to-vigorous PA (MVPA) per week for pregnant women with GDM [6–8], adherence rates remain suboptimal. For example, only 17.66% in China meet this target [9]. This discrepancy underscores the need to better understand the behavioral mechanisms influencing PA engagement in this population.

Self-control is defined as the ability to regulate individuals’ thoughts, emotions, and behaviors to overcome impulses, resist temptations, and delay immediate gratification [10, 11]. As a subset of self-regulation, which addresses the “how to do” through structured planning and the organization of personal resources, self-control specifically concerns whether to act when goal-directed behavior (e.g., exercising) conflicts with competing temptation [12, 13]. In this context, temptations are conceptualized as immediate impulses (e.g., the urge for prolonged sedentary rest) that offer instant comfort but conflict with the long-term health benefits of planned PA when PA is medically safe and feasible [14, 15]. Growing evidence suggests that self-control is consistently associated with PA engagement. For example, a meta-analysis involving 38,288 participants revealed that individuals with higher self-control were more likely to maintain higher PA levels [16].

Due to concerns for maternal and fetal health, pregnant women with GDM often report strong intentions to engage in PA and frequently set PA goals based on professionals’ recommendations [17, 18]. However, they frequently face competing temptations due to the combined effects of glycometabolism and pregnancy, such as favoring rest after dietary restrictions or choosing sedentary leisure over planned PA [19–21]. In such contexts, intention alone may be insufficient to ensure PA engagement, particularly when individuals must resolve temptation-goal conflicts under constrained physical, social, or contextual conditions [22, 23]. Existing research on PA determinants in pregnant women with GDM has primarily focused on factors within the COM-B framework, including capability (GDM knowledge, physical discomfort and fatigue), opportunity (lack of social support, environmental and time constraints), and motivation (fear of fetal harm, perceived health benefits) [17, 24, 25]. While this framework has provided valuable insights into barriers and facilitators of PA, it offers limited understanding of how individuals experience and respond to temptation-goal conflicts. Importantly, these processes are not isolated but are situated within the broader social and environmental systems that shape their daily life. Self-control may thus be understood as a process through which individuals attempt to manage such conflicts, operating within existing levels of capability, opportunity, and wider systemic constraints.

Therefore, this study conducted a qualitative study to explore how self-control processes are enacted by pregnant women with GDM within surrounding systems. The findings will enrich our understanding of self-control in the context of PA among pregnant women with GDM and inform the design of behavior change interventions that empower this population to integrate PA into daily life and increase PA levels.

## Methods

### Study design

A descriptive qualitative study was conducted to comprehensively and directly explore how pregnant women with GDM execute self-control to perform PA in their routines, as such design allows for in-depth description and analysis of a phenomenon, offering rich insights into participants’ subjective experiences when little is known about the current situation [26, 27]. Inductive qualitative content analysis was applied in this study, as it allows for the systematic and comprehensive identification of key concepts when existing knowledge about the phenomenon is limited or fragmented [28]. This study followed the standards for reporting qualitative research (SRQR) [29]. This study was approved by the Ethics Committee of Women’s Hospital, School of Medicine, Zhejiang University (approval number: IRB-20250119-R).

### Participants and setting

Participants were recruited using purposive sampling at a tertiary hospital in Hangzhou City, Zhejiang Province, China, from April to May 2025. The inclusion criteria included women who had been diagnosed with GDM based on a 75 g oral glucose tolerance test at least one week prior to the interview, had a singleton pregnancy, were medically suitable for MVPA, and had no language barriers or cognitive impairments. Exclusion criteria included a diagnosis of type 1 or type 2 diabetes mellitus, the presence of other pregnancy complications, or current participation in other clinical intervention trials targeting the PA.

During the recruitment phase, clinic nurses screened women against predefined inclusion criteria during routine antenatal visits. The first author (QL, a female PhD candidate in nursing with systematic qualitative research training) then invited eligible women, providing a brief explanation of study’s objectives and procedures. The maximum variation sampling technique was also applied to enhance sample heterogeneity across age groups (< 30, 30–35, > 35 years), gestational stages (second and third trimesters), PA levels (meeting or not meeting guideline). The sample size was determined by the principle of data saturation, which means that interviews were stopped after at least 10 participants and that no new information emerged in three interviews [30].

### Data collection

Face–to–face semi-structured interviews were conducted to collect data. The initial interview outline was developed through the literature review and consultation with experts in behavioral psychology, obstetrics, and obstetric nursing. After pilot interviews with three pregnant women with GDM, the final interview outline was determined without any modification (see supplementary material file 1). The data from these three interviews were also included in the final analysis. All participants received comprehensive pre-interview briefings, including purpose, significance, estimated duration, audio-recording procedures, and their rights to withdraw or decline questions at any time. Written informed consent was obtained prior to participation.

At the beginning of the interview, demographic and obstetric information, including age, educational attainment, monthly income, employment status, gestational age, parity, BMI, method of conception, and PA levels, was collected. Specifically, PA levels were assessed using the validated Chinese version of the pregnancy physical activity questionnaire (PPAQ) [31]. This 31-item instrument classifies activities by metabolic equivalent task (MET) intensity: sedentary (< 1.5 METs), light (1.5–2.9 METs), moderate (3–6 METs), and vigorous (> 6 METs). Weekly energy expenditure (MET-h/week) was calculated by multiplying the MET value of each activity by its duration weight. The PPAQ has good reliability and validity, with a content validity index of 0.94 and test-retest reliability ranging from 0.88 to 1.00. Consistent with guidelines and previous studies [7, 8, 32], a minimum of 150 min of MVPA per week (7.5 Met-h/week) was considered to meet the PA standard.

All interviews were conducted by the first author in a private room within the hospital to ensure a quiet and confidential environment. Although an interview outline was used as a reference, the interview process remained dynamic and flexible, with the order of questions adjusted based on the participant’s flow of conversation. The interviewer also used follow-up questions and prompts whenever necessary to encourage further elaboration. Typical prompts included asking participants to give a recent example (e.g., “Can you tell me about a recent time this happened?”), describe the situation and context (“What was happening around you at that moment?”), and elaborate on the self-control process (e.g., “What did you do next to help yourself follow through?”). Brief clarification probes (e.g., “What did you mean by…?”) and continuation prompts (e.g., “Then what happened?”) were also used. Observational notes, such as facial expressions, movements, and eye contact, were used to record the nonverbal cues.

### Data analysis

Inductive qualitative content analysis was used to analyze the qualitative data [28]. Within 24 h post-interview, two researchers (QL, FX) independently transcribed the audio recordings and observational notes. They then exchanged transcripts and cross-checked them against the recordings to verify accuracy. Discrepancies in transcription, such as the local dialectal expressions and segmentation that could change the intended meaning, were resolved through side-by-side comparison and discussion between the two researchers (QL, FX). If consensus could not be reached, the corresponding author (SF, a doctoral supervisor specializing in the field of PA for pregnant women, with extensive experience in qualitative research) served as a third reviewer, and all three researchers jointly listened to the original audio and reviewed both transcripts to finalize the wording. Each transcript served as a unit of analysis and was read repeatedly by two researchers (QL, FX) to gain a comprehensive understanding and achieve immersion before coding. Next, the text contents were coded with the explanations, which were then grouped into subthemes and themes on the basis of their interrelationships. These codes, subthemes, and themes were refined through weekly research team meetings, comprising members with experience in PA management for pregnant women with GDM, behavioral psychology, and qualitative research, until thematic saturation and consensus were achieved. This diversity in perspectives enabled comprehensive data interpretation and analytical rigor. Finally, the identified themes were checked with participants to confirm their interpretive validity, enhancing the credibility of the findings.

### Rigor

To enhance the rigor of this qualitative research, the elements of credibility, dependability, confirmability, and transferability for qualitative research recommended by Lincoln, and Guba [33] were followed. Credibility was supported through: (1) procedures to facilitate open interviews; (2) peer debriefing sessions with experts, research teams, and hospital administrators; (3) checking findings with participants; and (4) comprehensive audio-recording. Dependability was strengthened through supervision of the entire research process. For confirmability, independent coding of raw data by two researchers and transparent documentation of theme refinement were preformed until consensus was reached. Transferability was enabled through detailed descriptions of the study settings, eligibility criteria, recruitment process, and analysis procedures. All measures used to enhance rigor are detailed in supplementary material file 2.

## Results

### Participants’ demographic characteristics

A total of 24 participants were enrolled in this study. The mean interview length was 35 min (ranging from 28 to 55 min). The participants were aged 23 to 38 years (mean ± SD, 31.46 ± 3.95), with gestational ages ranging from 25^+ 1^ to 40^+ 6^  weeks. The majority of participants (75%) held a bachelor’s degree or above. Most were either employed or self-employed, with only four participants reporting no current employment (16.67%). 50% of the participants met the PA standard. The detailed demographic characteristics are shown in Table 1. To ensure the privacy of the participants, P1-P24 were used for identification.

**Table 1:** Participants characteristics

Participant	Age	Educational attainment	Monthly income	Employment status	BMI	Gestational age (week)	Parity	ART	Meet PA standard
P1^a^	34	Undergraduate	6000- < 9000	Private enterprises employee ^b^	20.89	37^+ 1^w	0	No	No
P2^a^	30	Junior college	6000- < 9000	Engaged in business	28.19	31^+ 4^w	0	Yes	Yes
P3^a^	27	Undergraduate	6000- < 9000	Not in employment	24.02	32^+ 2^w	0	No	No
P4	28	Undergraduate	≥ 9000	Private enterprises employee	25.97	33^+ 5^w	0	No	No
P5	33	Undergraduate	< 6000	Not in employment	21.63	31^+ 6^w	0	No	Yes
P6	30	Postgraduate	≥ 9000	Private enterprises employee ^b^	23.43	38^+ 5^w	0	Yes	Yes
P7	38	Undergraduate	6000- < 9000	Private enterprises employee	28.63	36^+ 2^w	1	No	No
P8	27	Postgraduate	≥ 9000	State - owned enterprises employee	23.05	25^+ 6^w	0	No	No
P9	30	Postgraduate	≥ 9000	Government employee	18.64	27^+ 0^w	0	No	Yes
P10	37	Junior high school	< 6000	Private enterprises employee ^b^	29.76	28^+ 6^w	2	No	Yes
P11	36	Undergraduate	≥ 9000	Private enterprises employee	20.64	25^+ 1^w	1	No	No
P12	31	Undergraduate	6000- < 9000	Private enterprises employee	23.61	28^+ 1^w	0	Yes	Yes
P13	30	Undergraduate	≥ 9000	Private enterprises employee	25.64	30^+ 2^w	0	No	Yes
P14	23	Undergraduate	< 6000	Not in employment	23.87	27^+ 1^w	0	No	No
P15	33	Junior college	≥ 9000	Private enterprises employee	27.64	29^+ 3^w	1	No	Yes
P16	26	Undergraduate	< 6000	Student	33.66	35^+ 5^w	0	No	No
P17	27	Senior high school	6000- < 9000	Independent work	25.00	40^+ 2^w	0	No	No
P18	33	Postgraduate	≥ 9000	State - owned enterprises employee ^b^	25.24	39^+ 5^w	1	No	Yes
P19	32	Junior college	< 6000	Private enterprises employee	30.84	30^+ 3^w	0	No	No
P20	38	Undergraduate	≥ 9000	Private enterprises employee	32.79	28^+ 5^w	1	No	Yes
P21	32	Undergraduate	≥ 9000	Private enterprises employee ^b^	23.36	40^+ 6^w	0	No	Yes
P22	31	Undergraduate	≥ 9000	Private enterprises employee	26.42	30^+ 3^w	0	No	No
P23	27	Specialized Secondary Schools	6000- < 9000	Not in employment	23.14	36^+ 0^w	1	No	Yes
P24	35	Postgraduate	≥ 9000	State - owned enterprises employee ^b^	23.74	39^+ 6^w	0	Yes	No

Note: *ART* Assisted reproductive technology, ^a^ pilot interviewing participants, ^b^ maternity leave, PA standard: MVPA ≥ 150 min/week

### Thematic overview of qualitative data

**Fig. 1:**
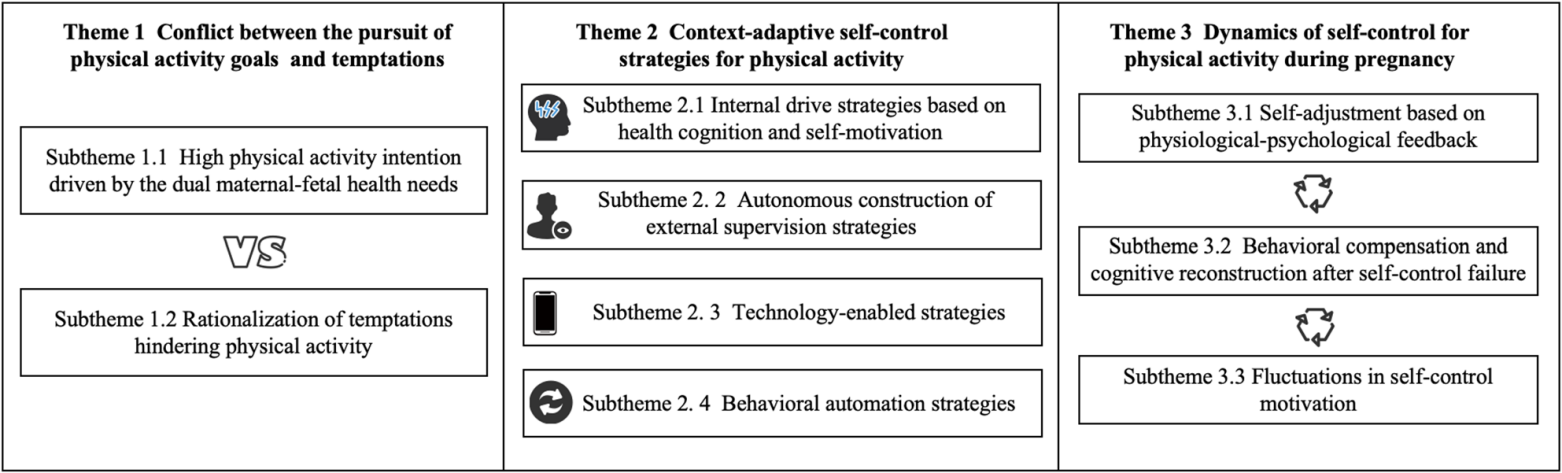
The framework of themes and subthemes. Note: Theme 1 describes the temptation-goal conflict in PA engagement, contrasting high PA intention (Subtheme 1.1) with rationalization of temptations that can reduce PA engagement (Subtheme 1.2). Theme 2 summarizes context-adaptive self-control strategies reported by participants (Subthemes 2.1–2.4). Theme 3 depicts the dynamic nature of self-control across pregnancy (Subthemes 3.1–3.3)

After analyzing the qualitative data, a total of three themes (conflict between the pursuit of PA goals and temptations, context-adaptive self-control strategies for PA, and dynamics of self-control for PA during pregnancy), with nine subthemes, were identified. The framework of the themes and subthemes is shown in Fig. 1.

#### Theme 1 Conflict between the pursuit of PA goals and temptations

##### Subtheme 1.1 High PA intention driven by the dual maternal-fetal health needs

Following GDM diagnosis, pregnant women proactively sought health information independently or received clinician counseling on GDM risks and PA benefits during antenatal check-ups. To achieve glycemic control, vaginal delivery, and prevent fetal macrosomia, these participants demonstrated strong PA engagement intentions and aimed to meet the PA level recommended by healthcare professionals.

P2: After diagnosis, I went to the nutrition clinic. The doctor gave me a meal plan and emphasized the need to increase PA. I thought I should control myself … what if I couldn’t have a natural childbirth?

P16: As a master’s student in traditional Chinese medicine, I absolutely know how crucial PA is for reducing GDM complications. Since I am unemployed now, I thought I should prioritize more PAs.

P23: According to my online search, 30 to 60 min of daily MVPA is recommended. My biggest worry was the baby growing too large, so I aimed to control my blood sugar.

##### Subtheme 1.2 Rationalization of temptations hindering PA

Despite strong intentions to engage in PA, pregnant women with GDM encountered multiple temptations during implementation. The desire to rest and sedentary social interaction, and high smartphone use emerged as core temptations disrupting their PA plans.

P1: I thought I was just lazy. I knew I needed to exercise, but pregnancy intensified my reluctance to move. I rarely forced myself to do PA; I predominantly chose sleep or smartphone use [laugh].

P8: If it hadn’t been for the high blood sugar problem caused by GDM, I probably wouldn’t have considered doing MVPA. Honestly, I just want to sit and scroll through my phone after work.

P19: During workdays, I walked around when I’ve sat too long. However, after work, once I lay down I don’t want to move. I am fascinated by mobile games, and I have to play every night before bed.

By assigning “necessary” meanings for temptations, some pregnant women with GDM framed them as adaptive responses to pregnancy-related physical and emotional demands. For example, they interpreted the desire to rest as “compensation for body burden during pregnancy”; they viewed sitting to chat with family as “meeting the emotional support needs of pregnancy”; and they described high smartphone use as “a necessary way to distract from boredom during pregnancy and adjust their mood”. Such rationalization was often accompanied by reduced PA engagement in daily life.

P4: I think pregnancy is tiring enough, and it’s easy to feel sleepy, so why do I need to push myself? Besides, I didn’t want to be alone. After getting pregnant, I felt more prone to mood swings. If my husband didn’t accompany me, I didn’t want to go out; I just wanted to sit at home and chat with him. I think many people are like me. It’s a normal emotional need, right?

P11: I don’t think I could control myself [laugh]. Subjectively, I know I need PA, but after a long day at work, I was exhausted and my body felt heavy, so I just thought, “Forget it, I’ve worked hard enough”. Sometimes I forced myself to go for a walk, but it felt like a chore.

P14: Doctors and family members all told me I need to control my weight and engage in PA, but apart from walking, I rarely moved. I lacked the willpower to do that. I often felt bored after pregnancy, so I usually played on my phone to distract myself. PA didn’t make me happy, and playing on my phone was better.

#### Theme 2 Context-adaptive self-control strategies for PA

##### Subtheme 2.1 Internal drive strategies based on health cognition and self-motivation

Some pregnant women employed cognitive strategies to counter temptation by consciously reflecting on potential harm to themselves and their fetuses. By imagining negative maternal and fetal outcomes for succumbing to rest, they reinforced their sense of health responsibility. Others increased their internal drive by envisioning positive future rewards, linking successful glucose management through PA to postpartum happiness.

P9: Sometimes, if I ate something high in sugar or stayed inactive all day, I felt guilty. I immediately moved those foods away and did more PA to compensate. I kept reminding myself that for a smooth delivery and good health, I need more PA…if I could have a natural birth, I might recover within two months postpartum and travel. During maternity leave, I could visit many places.

P21: I searched for health information and learned that GDM significantly increases future type 2 diabetes risk. A colleague developed diabetes after GDM. I’m only 30 years old. If I get diabetes, I might live with it for 50 years, which terrified me. Every time I wanted to lie down, I pictured my grandfather’s late stage of diabetes. That made me get up and walk immediately.

##### Subtheme 2.2 Autonomous construction of external supervision strategies

Some participants proactively informed family members of their PA plans and goals, transforming individual behavior into shared family responsibility to prevent yielding to temptations. Daily reminders and accompaniment from family members enhanced behavioral accountability.

P10: As an advanced maternal age pregnant woman, my metabolism wasn’t good, so I should do more PA. I asked my husband to supervise and remind me.

P22: I still sat a lot; walking was my main PA. I felt I needed my father’s help and routinely asked him to remind me to walk.

Some pregnant women indicated that they had participated in professional fitness or exercise guidance classes. Leveraging fixed schedules, timely instructor feedback, and peer modeling effects reduced the susceptibility to temptations during MVPA implementation. This approach of group supervision and professional feedback not only improved compliance but also alleviated loneliness and enhanced confidence.

P6: I usually only walked at home and avoided housework. I knew I needed more MVPA, but I couldn’t always motivate myself. Hospitals provide free yoga and pregnancy gymnastics classes. Once I made an appointment, I had to attend because missing several sessions would prevent me from booking again. This was quite effective. Exercising in a group was fun, and the teacher coached us, which was more effective than doing it alone at home.

P21: Being passive, I required supervision, so I enrolled in a prenatal yoga class. Mandatory weekly one-on-one sessions ensured attendance. The teacher adapted exercise plans to my pregnancy stage, eliminating safety concerns and enabling me to meet PA goals. I think external supervision is better.

##### Subtheme 2.3 Technology-enabled strategies

In the digital era, pregnant women with GDM could utilize smart applications’ algorithmic recommendations and real-time interaction functions to resist temptations and enhance self-control for PA. Some participants stated that they actively browsed GDM-related videos by relying on the personalized recommendation algorithms of applications, such as Rednote and TikTok, which triggered system content-pushing mechanisms. Sustained exposure helped them pay less attention to entertainment content, stimulated their PA intentions, and promoted PA engagement.

P3: When I was first diagnosed with GDM, I felt anxious and searched for health information. I repeatedly viewed related tips on Rednote, and the algorithm subsequently prioritized PA and pregnancy gymnastics videos. During that period, whenever I saw them, I would try to follow along. The same occurred on TikTok. Each app opening served as a PA reminder.

Moreover, some participants subscribed to live-streamed pregnancy gymnastics programs. Scheduled notifications of daily live broadcasts and real-time interactions helped counteract temptations, such as the urge to rest.

P13: I had previously attempted pregnancy gymnastics programs on TikTok. The daily live broadcasts and real-time comments made me feel like I belonged to a group. This structured group was motivating; alone, I would have chosen to rest.

P20: TikTok provides numerous live broadcasts of pregnancy gymnastics with fixed schedules. When I saw the notifications such as “Your followed live broadcast is now live”, I knew it was time to exercise.

##### Subtheme 2.4 Behavioral automation strategies

Some participants transformed deliberate PA into automated routines by integrating PA with personal interests and establishing fixed schedules. By gaining enjoyment and satisfaction from PA, this approach created a beneficial feedback chain that developed habitual PA and reduced susceptibility to temptation.

P9: Since six months before pregnancy, I have practiced yoga for 30 min daily, five days weekly. When I was diagnosed with GDM at 24 weeks, I felt devastated and believed that I needed more MVPA. I currently maintain 4–5 pregnancy gymnastics a week at a fixed time, mainly 3:30 − 5 PM after the afternoon snack. I rarely give up exercising because of temptations. Having sustained this long-term, I no longer perceive it as persistent but rather as an ingrained daily routine.

P10: While employed, daily PA felt obligatory. After leaving work, lying at home all day caused depression and emptiness. Constant eye interaction with elderly families without meaningful conversation became unbearable. I discovered that walks, housework, or pregnancy gymnastics improved my mood, transforming PA from a task into a habit.

P20: Daily dog walking constituted my additional PA. If someone is unable to push themselves to engage in PA, I suggested that they could adopt dogs to create mandatory walking routines, while shopping enthusiasts could go shopping more. Combining interests with PA reduces perceived effort and facilitates habit formation.

#### Theme 3 Dynamics of self-control for PA during pregnancy

##### Subtheme 3.1 Self-adjustment based on physiological-psychological feedback

Pregnant women with GDM dynamically adjusted PA duration and frequency according to their blood glucose and physical sensations. Moreover, when a single strategy proved ineffective, they actively alternated different self-control strategies to achieve PA goals.

P5: My primary PA motivation was glycemic control. I once walked almost 2,000 steps while grocery shopping in the morning without performing pregnant gymnastics. My blood sugar was high when I checked it later, which reminded me that walking alone was insufficient; I must do MVPA. Initial fatigue during pregnancy gymnastics gradually led to mood improvements, potentially as a result of dopamine release.

P7: I bought a yoga ball and placed it in my house in the belief that seeing it would inspire me to exercise, but it didn’t work. I also asked my husband to remind me, but he was more unreliable and lazier than me [laugh]. Later, I found focusing on hyperglycemia risk was more effective. I’ve been less focused on PA lately because diet management has kept my blood sugar stable.

P18: After being diagnosed with GDM, I closely monitored my PA level. Nevertheless, there were moments when I just wanted to sit and play with my phone or chat with friends. I monitor my blood sugar daily; if within normal limits, I may be sedentary; if not, I’ll engage in more MVPA.

##### Subtheme 3.2 Behavioral compensation and cognitive reconstruction after self-control failure

Some participants reported that they avoided self-blame after yielding to temptations and skipping PA by downplaying the significance of failure. They prevented the “broken windows effect” through compensatory PA behavior.

P12: I followed pregnancy gymnastics videos on Rednote, but sometimes I got distracted by phone notifications and stopped exercising to play on my phone. When I noticed this happening, I’d quickly do some MVPA right then. Or if I’d been lazy all day and barely moved, I’d do 20 min of yoga before bed. It’s normal to slip up sometimes; what matters is remembering to perform PA daily. You can’t be active all the time, but you also shouldn’t quit completely just because you missed one day.

##### Subtheme 3.3 Fluctuations in self-control motivation

Some pregnant women reported initial efforts to overcome temptations following GDM diagnosis. However, maintaining PA before habit formation required substantial effort, leading to a tendency to abandon PA. Additionally, some participants indicated that poor blood glucose improvement gradually weakened their self-control motivation to overcome the temptation, eventually leading to PA discontinuation. Some participants also increased their PA frequency and intensity in late pregnancy to prepare for delivery.

P3: I did try to engage in PA more before but didn’t persist. It felt like PA didn’t help my blood sugar and just wore me out, so I quit forcing myself. Now I just stand up when I’ve been sitting too long.

P24: I know I need PA and mean to do it, but whether I actually do it is another story [laugh]. After my diagnosis, I went to one pregnancy gymnastics class that was supposed to help with natural delivery. However, it felt like a chore, and I was exhausted as I needed to remind myself to engage in PA. Now I’m near my due date, and since the doctor says more PA in late pregnancy benefits delivery, I’ve increased PA levels.

P21: These days, since I’m beyond 40 weeks of pregnancy with no symptoms of labor, I’m doing PA more vigorously. I hope to have a natural delivery.

## Discussion

This qualitative study explored how self-control processes may explain the PA intention-behavior gap among pregnant women with GDM in the physical and social contexts. Despite strong PA intentions, sustained engagement was challenged by conflicts between PA goals and temptations, with rationalization of temptation often accompanying PA disengagement. To address these conflicts, participants employed context-adaptive self-control strategies, including internal drive strategies based on health cognition and self-motivation, autonomous construction of external supervision strategies, technology-enabled strategies, and behavioral automation strategies. Crucially, self-control processes were dynamic throughout pregnancy.

PA maintenance is a complex and dynamic process involving reflective, regulatory and reflexive mechanisms from intention formation to sustained behavior [34]. This study showed that while many pregnant women with GDM reported PA intentions, they commonly encountered PA temptation-goal conflicts. Self-control can support the identification and regulation of temptations during goal pursuit [35, 36]. According to the two-stage model of self-control [37], when faced with temptation, individuals will draw on self-control strategies to promote goal-pursuit only after they have identified temptation-goal conflict. Our findings suggested that this conflict identification step can be weakened when competing temptations (e.g. the urge to rest, sedentary social interaction, and high smartphone use) are framed as necessary during pregnancy. In these moments, the temptations become less experienced as a conflict with PA goals. Such interpretations may be amplified by GDM-related physical fatigue, edema, and disease-related emotion [38, 39], thereby weakening conflict identification and making it less likely that self-control strategies are initiated. As a cognitive defense mechanism, rationalizing may alleviate the discomfort associated with unmet PA goals [40]. However, persistent reliance on rationalization may make resting or other sedentary options feel more acceptable, which can reduce the likelihood of following through with planned PA when PA is medically safe and feasible [41]. This perspective highlights how psychological coping processes interact with contextual constraints, which may help explain why COM-B elements alone may not fully explain PA levels in pregnant women with GDM. Therefore, healthcare professionals could provide a compassionate way to understand their difficulties and support reflective awareness of temptation-goal conflicts.

Individuals employ diverse self-control strategies to resist or avoid temptations among different health behavior domains [42, 43]. This study identified four types of self-control strategies enabling pregnant women with GDM to overcome temptations and achieve PA goals. These strategies can be seen as a dynamic interaction of internal driving force system (such as internal drive strategies based on health cognition and self-motivation), social environment support system (such as autonomous construction of external supervision strategies, technology-enabled strategies), and behavioral automation system (such as behavioral automation strategies).

First, pregnant women with GDM enhance intrinsic PA motivation by reflecting on the harms of giving in to competing temptations for themselves and their fetuses, and envisioning a better life after successful blood glucose control and delivery. These findings are consistent with prior evidence that self-control is correlated with stronger utility belief of health behavior and reflects the value-based nature of self-control [44, 45]. Thus, healthcare providers may support this process by discussing potential risks of inactivity alongside the benefits and safety of PA, and by helping pregnant women with GDM set personally achievable PA goals.

Second, this study revealed that pregnant women with GDM actively shape their social environment to promote self-control. These findings align with Milosevic et al. [46], who reported that seeking social support is a crucial component of people’s repertoire of self-control strategies across health behaviors. These strategies involved establishing supervision systems, triggering app content-drive mechanisms, and participating in regular gymnastics live broadcasts. By transferring self-control demands to social accountability and embedding PA behavioral cues in daily routines, these strategies can be seen as a way of deliberate environmental selection and modification to align with PA goals. Such structuring minimizes the cognitive effort required for sustained self-control [47, 48]. Moreover, digital technologies amplify these effects by facilitating the construction of supportive social environments [49]. Features such as scheduled notifications of daily live broadcasts, real-time interactive broadcasts, and personalized algorithmic recommendations play both supervisory and motivational roles. Therefore, interventions should focus on enhancing awareness of the proactive environment shaping and encouraging pregnant women with GDM to leverage the digital technologies. For example, healthcare providers could encourage them to design contextual digital reminders such as post-meal PA notifications and provide them with an app that offers tailored PA content recommendations.

Furthermore, the findings indicated that pregnant women with GDM gradually develop habitual PA behavior by integrating it with their personal interests and performing it at fixed times, which helps them be less susceptible to temptation. Gillebaart & Schneider [50] indicated that habits play an important role in enabling successful yet effortless self-control, helping individuals better solve temptation-goal conflicts without struggle. By building strong habitual behavior, pregnant women with GDM are able to perform PA without temptation blocks and conscious effort, which is also the ultimate goal of PA maintenance [35, 51]. The PA habit is formed through repeated execution of behaviors in the presence of consistent cues or contextual features [52, 53]. Therefore, healthcare providers could encourage pregnant women with GDM to perform PA at fixed times and in preferred settings, fostering the development of habitual behavior through enjoyable routines.

The findings also indicated that the self-control for PA among pregnant women with GDM is a dynamic process. It involves continuous feedback-driven goal adjustment, strategies switching, failures coping, and motivational fluctuation management. This finding aligns with the extended process model of self-control by Werner et al. [14], which emphasizes the monitoring process of self-control determining whether to maintain, switch, or stop regulatory goals and strategies. Unlike individuals with stable chronic conditions, pregnant women with GDM face the dual challenges of blood glucose fluctuations and gestational discomfort. This situation creates a complex and specific nature of PA goal pursuit that need continuous self-control adjustments. This study found that glycemic control appeared to act as a monitoring cue that informed participants’ decisions about when and how much PA to do. Some participants described that when their glucose was within target ranges through dietary management, they perceived less need to add further PA at that time. This interpretation may reflect an adaptive self-control process. However, it may also reduce the significance of PA goals and make the desire to rest feel more acceptable, which could align with rationalization of temptations in certain situations. Moreover, a decrease in the complexity of the glucose time series index, an index measuring blood glucose homeostasis, is associated with impaired executive function and attention [54], which are considered as components of self-control [55]. These findings indicate that self-control is malleable and shaped by metabolic, physical, and psychosocial factors. Therefore, interventions could focus on training pregnant women with GDM to manage self-control resources, such as prioritizing strategies requiring less cognitive effort. Moreover, real-time metabolic feedback apps could also be provided for pregnant women with GDM to support adaptive goal setting.

Several limitations of this study should be acknowledged. First, although our findings suggest that self-control strategies encompass both individual and environmental dimensions, we did not explicitly analyze the wider social, ecological, cultural, and healthcare system determinants of PA. Future research should explore these systemic factors and how they intertwine with self-control to shape opportunities and constraints for PA engagement. Second, participants were recruited exclusively from Hangzhou City, Zhejiang Province, where relatively strong medical and PA infrastructure may facilitate PA engagement. Although maximum variation sampling was employed, most participants were employed, highly educated, and primiparous. These characteristics may be associated with relatively greater schedule flexibility, higher health literacy, and lower child caregiving demands, potentially making self-control for PA more feasible in this sample than in the broader population. Future research is needed to validate and extend the identified dynamic self-control processes in more diverse sample and context. Third, it is possible that some effective self-control strategies for managing temptations were not captured in this study. Future research would benefit from exploring a broader range of strategies. Finally, the qualitative nature of this study makes it difficult to quantify the relative effectiveness or prioritization of different self-control strategies for PA among pregnant women with GDM. Future studies could adopt a mixed-method approach that integrates the in-depth mechanism analysis of qualitative research and the data verification of quantitative research. This could determine which strategies might be more or less beneficial in different gestational periods, thereby providing more comprehensive support for the formulation of personalized self-control interventions.

## Conclusions

This descriptive qualitative study explored how pregnant women with GDM navigate self-control to engage in PA, offering a self-control perspective on the intention–behavior gap research and PA enhancement among this population. This study highlights that the rationalization of temptation could coincide with reduced PA engagement, framing competing temptations (e.g., the urge to rest) as “necessary” in the context of pregnancy. Future interventions to support sustained PA engagement among this population could aim to help women identify and challenge rationalizing thoughts by strengthening goal awareness. Such interventions might also incorporate individualized self-control strategies tailored to specific pregnancy stages, leverage mHealth platforms for customized reminders, and encourage fixed time PA routines based on interest to foster habit formation.

## Supplementary Information

Supplementary Material 1.

Supplementary Material 2.

## Data Availability

The datasets used and/or analysed during the current study are available from the corresponding author on reasonable request.

## Abbreviations

GDM: Gestational diabetes mellitus
PA: Physical activity
MVPA: Moderate-to-vigorous physical activity
WHO: World Health Organization
PPAQ: Pregnancy physical activity questionnaire
MET: Metabolic equivalent task
