# Supplementary material for: Bridging the intention-behavior gap in physical activity among pregnant women with gestational diabetes mellitus: a qualitative study of self-control strategy use and dynamics
Source: Int J Behav Nutr Phys Act. 2026 Apr 20;23:57. doi: 10.1186/s12966-026-01923-y (PMC13227627; doi:10.1186/s12966-026-01923-y)
Supplement: Supplementary file 1 — Supplementary Material 1. [file 12966_2026_1923_MOESM1_ESM.docx]

**Supplementary material file 1. The interview outline**

| Questions: | Rationale and Connection to Research Objective: |
| --- | --- |
| Since the diagnosis of gestational diabetes mellitus (GDM), what kinds of physical activity (PA) have you engaged in? | This opening question was designed to understand participants’ current PA status following GDM diagnosis and to create a comfortable entry point into the interview. It provides baseline information on participants’ PA engagement, which is essential for interpreting subsequent interview questions related to self-control experience. |
| Could you describe an experience where you felt tempted to give up PA? What happened at that time? | Experiencing PA goal-temptation conflict is a necessary trigger for the activation of self-control processes [1]. This question was designed to understand how pregnant women with GDM perceive, identify, and experience temptations that challenge their intention to engage in PA. It addresses the research objective of understanding when and why self-control is initiated or not. |
| When facing such temptations, what strategies do you use to control yourself and maintain PA? Why do you choose these strategies? | Once a conflict between temptation and a PA goal is identified and individuals are motivated to exert self-control, they may select and implement self-control strategies to regulate temptation and engage in PA[2]. This question addresses the research objective of understanding how pregnant women with GDM choose and apply specific self-control strategies when they are facing temptation. |
| Have you ever tried any methods to preemptively avoid temptations in order to facilitate PA? | In addition to regulating temptations as they arise, researches indicate that individuals may also engage in proactive efforts to prevent conflicts between temptation and PA goals before they occur [2 - 4]. This question addresses the research objective of understanding how pregnant women with GDM choose and apply proactive self-control strategies to reduce exposure to temptation. |
| During the process of avoiding or overcoming temptations to engage in PA, did you encounter any difficulties? How did you adjust and overcome them? | Self-control involves continuous self-monitoring and adaption. Individuals assess the effectiveness of their actions and flexibly decide whether to maintain, change, or abandon their goals and strategies [5]. This question addresses the research objective of exploring how pregnant women with GDM adapt their self-control efforts to maintain PA throughout pregnancy. |

**References**

1. Kotabe HP, Hofmann W. On Integrating the Components of Self-Control. Perspect Psychol Sci. 2015;10:618–38. https://doi.org/10.1177/1745691615593382

2. Werner KM, Ford BQ. Self‐control: An integrative framework. Social &amp; Personality Psych. 2023;17:e12738. https://doi.org/10.1111/spc3.12738

3. de Ridder D. Can self-control make you happy? Curr Opin Psychol. 2024;60:101875. https://doi.org/10.1016/j.copsyc.2024.101875

4. Duckworth AL, Gendler TS, Gross JJ. Situational Strategies for Self-Control. Perspect Psychol Sci. 2016;11:35–55. https://doi.org/10.1177/1745691615623247

5. Inzlicht M, Legault L, Teper R. Exploring the Mechanisms of Self-Control Improvement. Curr Dir Psychol Sci. 2014;23:302–7. https://doi.org/10.1177/0963721414534256
