# Supplementary material for: Bridging the intention-behavior gap in physical activity among pregnant women with gestational diabetes mellitus: a qualitative study of self-control strategy use and dynamics
Source: Int J Behav Nutr Phys Act. 2026 Apr 20;23:57. doi: 10.1186/s12966-026-01923-y (PMC13227627; doi:10.1186/s12966-026-01923-y)
Supplement: Supplementary file 2 — Supplementary Material 2. [file 12966_2026_1923_MOESM2_ESM.docx]

| **Quality criteria** | **Action taken by the researchers** |
| --- | --- |
| Credibility | Researchers explained confidentiality and the voluntary nature of participation, clarified that involvement would not affect clinical care, and emphasized that participants could skip questions or stop the interview at any time. The interview outline was confirmed after pilot interviews to improve clarity and sensitivity. During interviews, researchers used active listening, allowed pauses, and used open-ended and follow-up prompts to give participants space to elaborate on their experiences. |
|  | Peer debriefing sessions were conducted at multiple stages: prior to data collection, multidisciplinary consultations involving behavioral psychologists, obstetricians, obstetric nurses, and the research team were held to develop the initial interview guide, addressing substantive, methodological, legal, and ethical considerations. During data collection, weekly debriefings with hospital administrators, first author, and corresponding author addressed challenges in participant recruitment. During data analysis, weekly research team meetings facilitated the iterative refinement of subthemes and themes until consensus was reached. |
|  | Anonymized interview summaries, themes and subthemes, interpretive frameworks, and preliminary conclusions were shared with participants for confirmation to ensure that our interpretations captured their intended meanings. |
|  | For referential adequacy, all interviews were audio-recorded. Interview transcripts and observational notes were retained as raw data to enable independent verification of data accuracy and support the auditability of findings. |
| Dependability | The corresponding author supervised the entire research process to ensure methodological consistency and procedural traceability. |
|  | Two researchers independently transcribed audio recordings and field notes into textual format, conducted cross-checked verification for transcription accuracy, and resolved discrepancies through consultation with the corresponding author when required. |
| Confirmability | Codes were independently generated by two researchers based on raw data (e.g., interview transcripts and observational notes). |
|  | Weekly meeting documentation captured the iterative process of subthemes and themes refinement. |
|  | Data analysis followed the guidelines of inductive qualitative content analysis to maintain methodological objectivity. |
| Transferability | Purposeful sampling (for maximum variation in age, gestational weeks, parity, and PA levels) was implemented to strengthen the transferability potential of research findings. |
|  | A detailed description of the study settings, participant eligibility criteria, recruitment process, participant demographics, data collection, and data analysis procedures was comprehensively provided. |

**Supplementary material file 2. Action taken by the researchers to enhance rigor**
